# A Metasurface Plasmonic Analysis Platform Combined with Gold Nanoparticles for Ultrasensitive Quantitative Detection of Small Molecules

**DOI:** 10.3390/bios13070681

**Published:** 2023-06-27

**Authors:** Taohong Zhou, Weihao Ji, Hongli Fan, Li Zhang, Xugang Wan, Zhiyong Fan, Gang Logan Liu, Qingzhi Peng, Liping Huang

**Affiliations:** 1 Hubei Provincial Institute for Food Supervision and Test, Wuhan 430075, China; 2 College of Life Science and Technology, Huazhong University of Science and Technology, Wuhan 430074, China; 3 Key Laboratory of Detection Technology of Focus Chemical Hazards in Animal-Derived Food for State Market Regulation, Wuhan 430075, China; 4 Hubei Provincial Engineering and Technology Research Center for Food Quality and Safety Test, Wuhan 430075, China; 5 Liangzhun (Wuhan) Life Technology Co., Ltd., 666 Gaoxin Avenue, Wuhan 430070, China

**Keywords:** competitive immunoassay, gold nanoparticles, nanoplasmonic biosensor, small-molecule detection, sulfamethazine

## Abstract

Food safety related to drug residues in food has become a widespread public concern. Small-molecule drug residue analysis often relies on mass spectrometry, thin-layer chromatography, or enzyme-linked immunosorbent assays (ELISA). Some of these techniques have limited sensitivity and accuracy, while others are time-consuming, costly, and rely on specialized equipment that requires skilled operation. Therefore, the development of a sensitive, fast, and easy-to-operate biosensor could provide an accessible alternative to conventional small-molecule analysis. Here, we developed a nanocup array-enhanced metasurface plasmon resonance (MetaSPR) chip coupled with gold nanoparticles (AuNPs) (MSPRAN) to detect small molecules. As sulfamethazine drug residues in poultry eggs may cause health issues, we selected this as a model to evaluate the feasibility of using MSPRAN for small-molecule detection. The MSPRAN biosensor employed competitive immunoassay technology for sulfamethazine detection. The limit of detection was calculated as 73 pg/mL, with sensitivity approximately twice that of previously reported detection methods. Additionally, the recovery rate of the biosensor, tested in egg samples, was similar to that measured using ELISA. Overall, this newly developed MSPRAN biosensor platform for small-molecule detection provides fast and reliable results, facile operation, and is relatively cost-effective for application in food safety testing, environmental monitoring, or clinical diagnostics.

## 1. Introduction

With economic improvements, food safety awareness has increased worldwide. However, food safety incidents continue to occur frequently and thus stricter testing and technical requirements are called for by regulatory authorities [1,2]. Many food safety issues are caused by small-molecule drug residues and mycotoxins that can have a negative impact on human health and the environment [3,4]. Because of insufficient monitoring and research, antibiotic residues frequently contaminate eggs and pose health challenges [4]. Sulfamethazine is one of the commonly used antibiotic drugs in poultry breeding. The excessive use of sulfamethazine can leave drug residues in food [5], and their residues in poultry eggs may cause health issues, such as allergic reactions, destruction of the hematopoietic system, and emergence of drug-resistant strains [5,6].

Currently, the methods most commonly used to monitor small molecules include liquid chromatography–mass spectrometry [7], enzyme-linked immunosorbent assay (ELISA) [8], and thin-layer chromatography (TLC) [9]. These methods can be expensive, use large and complex equipment that require skilled operation, are time-consuming, or have limited sensitivity [10,11], potentially preventing their broad application in point-of-care diagnostics and private laboratories. Consequently, a portable, easy-to-operate, low-cost, fast, and highly sensitive biosensor for small-molecule monitoring during food supervision, environmental screening, and early diagnosis is required.

Surface plasmon resonance (SPR) presents an attractive tool for quantitative biomolecule analysis because of its low-cost, rapid, sensitive, and high-throughput detection [12]. SPR technology can monitor a molecule’s reactivity by measuring refractive index (RI) changes on the surface of the chip [1,13]. SPR, which uses optical biosensing, has been used for macromolecule analysis, based on mass changes on a chip. However, to use this approach for small-molecule detection, physical or biochemical technology that can improve the sensitivity of SPR would be required. For example, Arsenin et al. designed graphene oxide linking layers, which showed 30-fold higher sensitivity than that of carboxymethylated dextran layers [14]. Given that traditional SPR technology is widely implemented due to its large volume, the metasurface plasmon resonance (MetaSPR) is expected to become a tool for point-of-care detection [15,16]. Compared with traditional SPR, the collective oscillation of electron gas can be excited by light, without the need for another coupled device because of the periodic nanostructures used in MetaSPR [15,16]. Recently, metaSPR has been used for macromolecule and virus detection, such as detection of viral nucleic acids, C-reactive protein, and SARS-CoV-2 pseudovirus [15,16,17]; however, it has not yet been used for small-molecule detection. MetaSPR detection is performed directly for macromolecules based on mass changes detected on a chip, but it must be coupled with nanoparticles to analyze small molecules with a low molecular mass [18].

Gold nanoparticles (AuNPs) are attractive for biosensor development due to their unique physicochemical properties, such as their optical properties, biocompatibility, high molecular mass, high stability, antibody immobilization capabilities, and large specific surface area [19,20]. Due to the mass effect and electron coupling of AuNPs, they can be used as a signal amplification strategy in small-molecule detection by SPR [21]. When the frequency between the incoming light and the electrons of monodispersed AuNPs match, it could result in a localized SPR phenomenon where electron coupling between AuNPs and the MetaSPR chip occurs. This may enhance the MetaSPR signal [22]. Moreover, because of their high molecular mass and ability to immobilize antibodies, AuNPS can produce a dual-signal amplification effect [23]. AuNPs coupled with MetaSPR can be used for small-molecule detection based on a competitive immunoassay, where a small-molecule antigen immobilized on the chip competes with a small molecule in the buffer for binding sites on antibody-labeled AuNPs.

Here, we developed a MetaSPR chip coupled with AuNPs (MSPRAN) for rapid, low-cost, and high sensitivity small-molecule detection. We selected sulfamethazine as the model analyte to evaluate the capacity of the MSPRAN biosensor platform and applied a competitive immunoassay, where AuNP-labeled antibodies served as recognition units for antigens labeled on the MetaSPR chip, while sulfamethazine acted as an inhibitor. To evaluate the feasibility of this detection system, whole eggs were spiked with a sulfamethazine standard and the detection limit and recovery rate of this system were compared with those of enzyme-linked immunosorbent assays (ELISAs). The developed MSPRAN biosensor platform may provide an alternative means for small-molecule detection during food testing, environment monitoring, and early disease diagnosis.

## 2. Materials and Methods

### 2.1. Reagents

Trisodium citrate and tetrachloroauric acid (HAuCl4) were obtained from Nanjing Chemical Reagent Co., Ltd. (Nanjing, China). Tris hydrochloric acid solution (tris) and phosphate-buffered saline (PBS) were purchased from Sigma-Aldrich (St. Louis, MO, USA). Sulfamethazine, chloramphenicol, enrofloxacin, ofloxacin, florfenicol, and fipronil were obtained from R-Biopharm Rhone (Darmstadt, Germany). Sulfamethazine antibodies and antigens were obtained from Guangzhou youkangduo Biotechnology Co., Ltd. (Guagnzhou, China). A sulfamethazine ELISA kit was purchased from MLBIO Biotechnology Co., Ltd. (Shanghai, China). All chemicals were of analytical grade and required no further purification.

### 2.2. Fabrication of the MetaSPR Chip and Biosensor

The replica-molding technique was used for biosensor fabrication according to the methods described in our previous reports [24,25]. Firstly, the mold was photo- and ion-etched to produce a tapered nanocup array on a silicon wafer containing nanopillars with a diameter, depth, and period of 200, 450, and 300 nm, respectively. Following this, the mold was made hydrophobic using hexylsilane, dried in a vacuum dryer for 12 h, and then a layer of UV-curable polymer solution was applied. A polyethylene terephthalate sheet was placed on top, followed by UV-irradiation for 5 min. The polyethylene terephthalate sheet was then peeled off to form a polymeric nanocone array structure. The MetaSPR chip was mass-produced by depositing multiple layers of metal (15 nm titanium, 70 nm silver, and 20 nm gold layers) using electric spray technology. Finally, the biosensor was fabricated by cutting the MetaSPR chip into 12.4 cm × 8.3 cm sheets, which were affixed to a standard open-bottom 96-well plate, made using a 3D printer (Object 30 primer™; Stratasys Ltd., Rehovot, Israel). The physical and optical properties of the MetaSPR chip were recorded using a smartphone camera, scanning electron microscope, and a generic microplate reader.

### 2.3. Synthesis of AuNP and Labeled Antibodies

Redox reactions were performed to synthesize colloidal AuNPs using a previously reported method [26]. HAuCl4 (300 mL, 1 mM) was heated to boiling point, whereafter 15 mL of 75 mM citrate were added. The mixture was boiled for another 15 min, resulting in a dark red colloidal AuNP solution, and was then cooled to room temperature. The physical properties of AuNP solution were characterized by transmission electron microscopy. The sulfamethazine antibody was immobilized onto the surface of AuNPs using a previously described method [12,27]. Briefly, 1.5 mL colloidal AuNP solution was adjusted to pH 7.4 with 1 M tris (pH 9.27), followed by the addition of different concentrations of sulfamethazine antibodies (final concentrations 0.38 μg/mL, 0.76 μg/mL, 1.52 μg/mL, and 2.28 μg/mL) in 10 mM PBS (pH 7.4) to optimize the labeled AuNP density, and the mixture was incubated for 15 min. Then, 150 µL 10% bovine serum albumin (BSA) was used to block the AuNPs for 15 min, followed by centrifugation at 5400× *g* for 20 min. The supernatant was removed, and the precipitate was resuspended in R2 buffer (20 mM Tris (pH 7.3), 0.3% sucrose, and 0.05% PEG 20000), which was then stored at 4 °C until further use. In addition, solutions with different antibody labeling densities were evaluated to distinguish between a blank (basic buffer, 20 mM tris, 20 mM EDTA, 0.5% Tween-20) and a sample containing 1 ng/mL sulfamethazine, to determine the optimal labeling density.

### 2.4. Functionalizing the Surface of the MetaSPR Chip

A self-assembly process was adopted for forming carboxyl groups on the surface of the MetaSPR chip to immobilize sulfamethazine antigens. Each chip well was filled with 3-mercaptopropionic acid (50 μL, 50 mM) and left at 37 °C for 30 min, whereafter the wells were washed twice with isopropanol and deionized water. For immobilization, an 1-ethyl-(3-dimethylaminopropyl)-carbodiimide hydrochloride/N-hydroxysuccinimide mixture (50 μL, 400 mM/100 mM) was used to activate the carboxyl groups on the surface of the chip and then placed in 2-morpholinoethanesulphonic acid (pH 5.0) for 5 min at room temperature. Then, 50 μL of 20 μg/mL sulfamethazine antigens were added to the chip wells and left at 37 °C for 15 min. Subsequently, the chip wells were blocked with 130 μL of 1% BSA in PBS and incubated for 30 min at 37 °C. Thereafter, the liquid in the chip wells was discarded. The 96-well biosensor was subsequently dried in an oven at 37 °C for 8 min, sealed, and stored at 4 °C until further use.

### 2.5. Measurement of Small Molecules Using the MSPRAN Biosensor

A competitive immunoassay method was used for small-molecule detection. Sulfamethazine (50 μL, concentrations ranging from 0 to 32 ng/mL) diluted in R1 buffer (20 mM tris, 20 mM EDTA, 0.5% TW20, 1% BSA) was added to the immobilized sulfamethazine antigens in each chip well of the biosensor. The starting point of the reaction was measured using a generic microplate reader at full wavelength (500–700 nm). Then, 5 μL aliquots of AuNP-labeled sulfamethazine antibodies were added to different concentrations of sulfamethazine solution in each chip well, followed by incubation for 10 min at 37 °C in a thermostatic metal bath with shaking (7000 rpm). The terminal point of the reaction was then measured, and the output signal was obtained by subtracting the starting point value from the terminal point.

### 2.6. Small-Molecule Detection in Whole Egg

To verify the capability of the 96-well MSPRAN biosensor to detect small molecules, sulfamethazine-spiked whole eggs were chosen as a real-world sample. Unspiked eggs were used as a control. For spiked sample preparation, 10 mL acetonitrile, 2 g sodium chloride, 3 g anhydrous sodium sulfate, and different final concentrations of sulfamethazine (2 ng/mL, 4 ng/mL, and 16 ng/mL) were mixed with 5 g homogenized egg. The mixture was then centrifuged at 2500× *g* for 4 min, whereafter 2 mL of the supernatant were collected and incubated with 300 mg anhydrous magnesium sulfate, 100 mg N-propylethylenediamine, 100 mg C18, and 15 mg graphitized carbon black for 30 s with shaking. After incubation, the mixture was centrifuged at 2500× *g* for 4 min, and 1 mL supernatant was collected for nitrogen drying. The precipitate was redissolved in 500 μL R1 buffer and used for sulfamethazine detection. To compare accuracy, both the MSPRAN biosensor and an ELISA kit were used.

## 3. Results and Discussion

### 3.1. Principle of Small-Molecule Detection Using the MSPRAN Biosensor

**Figure 1:**
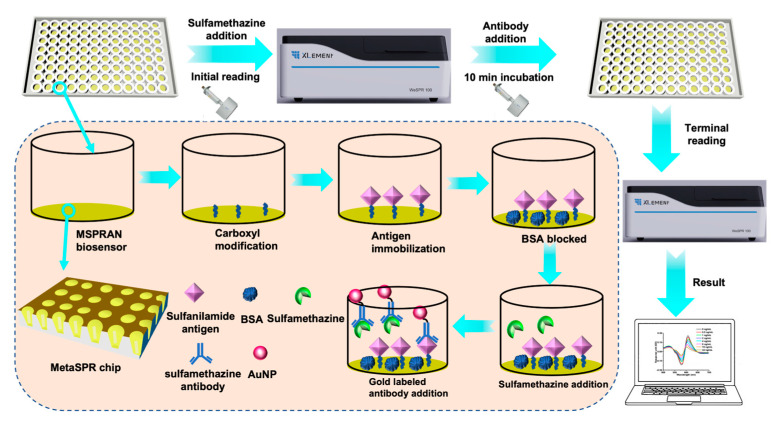
Setup of the competitive immunoassay used for small-molecule detection with the MetaSPR chip coupled with gold nanoparticles (AuNPs) (MSPRAN) biosensor.

When evanescent waves of incident light are coupled with a surface plasmon wave, they begin to oscillate, generating an SPR effect [28]. Due to the periodic nanocup structures of the MetaSPR chip, the SPR effect is produced without the need for another coupling element [16]. The MetaSPR chip consisted of multiple metal layers (15 nm titanium, 70 nm silver, and 20 nm gold layers), among which titanium ensured adhesion between the noble metal and chip substrate, while silver and gold allowed for good plasmon resonance [24,29,30]. Since molecule analysis with the MetaSPR was based on mass transfer, the biosensor required the assistance of other materials. AuNPs served as an amplification tool because they can significantly shorten analysis times due to their coupling effect and high molecular mass [12,31]. The recognition function of AuNPs conjugated to antibodies is mediated by noncovalent adsorption mechanisms, such as ionic, electrostatic, and hydrophobic interactions [32]. Considering this, a competitive immunoassay for small-molecule detection using the MSPRAN biosensor coupled with the AuNP amplifier was developed (Figure 1). In this method, small molecules in solution compete with antigens immobilized on the surface of the MetaSPR chip for binding sites on antibody-labeled AuNP. With an increase in small-molecule concentration, the amount of antibody-labeled AuNPs binding to antigens immobilized on the chip decreased, resulting in weak signaling.

### 3.2. Characterization of MetaSPR Chip and AuNPs

**Figure 2:**
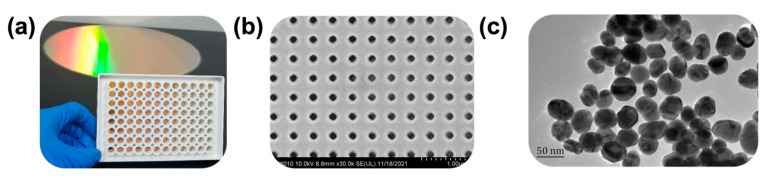
Uniformity characterization of a MetaSPR chip and gold nanoparticles (AuNPs). (**a**) Photograph of the MetaSPR chip and a 96-well MSPRAN biosensor. (**b**) Scanning electron micrograph of the MetaSPR chip (top view). (**c**) Transmission electron micrograph of AuNPs.

According to the image obtained with the camera of a smartphone, the chip showed a high optical response with a rainbow-like color (Figure 2a), indicating high sensitivity. Scanning electron microscopy of the top view of the chip showed a highly uniform nanocone array (Figure 2b), indicating that batches differed little and that the chips had high repeatability for mass production. In addition, the transmission electron micrograph showed a uniform size distribution of AuNPs (Figure 2c), indicating that particle size may have little influence on small-molecule detection.

**Figure 3:**
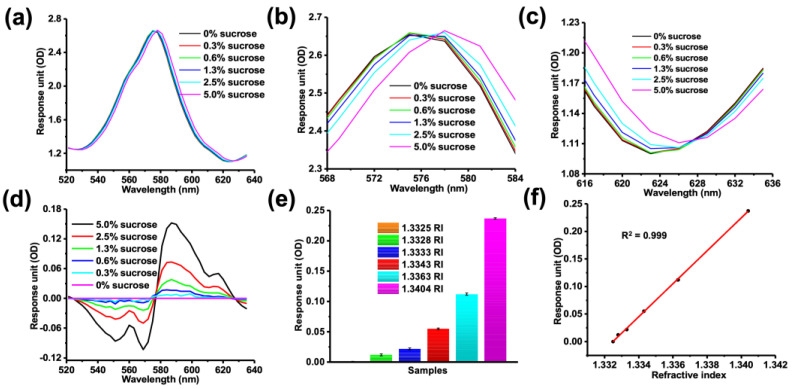
Sensitivity characterization of MetaSPR chip by measuring different concentrations of sucrose. (**a**) The full absorption spectra of sucrose at different concentrations (0–5%) with refractive index (RI) values ranging from 1.3325 to 1.3404. (**b**) Expanded view of the peak wavelength shift in panel (**a**) between 568 and 584 nm. (**c**) Expanded view of the shift in wavelength trough in panel (**a**) between 616 and 636 nm. (**d**) Changes in the two-wavelength optical density (OD) values (608 nm–581 nm) with different RIs (1.3325–1.3404). (**e**) Histogram of two-wavelength OD values showing changes at different RIs corresponding to varying concentrations of sucrose. (**f**) The linear relationship between two-wavelength OD values and different RI values (R^2^ = 0.999).

To further evaluate the sensitivity of the MetaSPR chip, different concentrations of sucrose solutions (0% to 5%, corresponding to refractive index (RI) values of 1.3325–1.3404) were used to measure absorption spectra with a generic microplate reader. The MetaSPR was shown to be highly sensitive, because it could measure an RI change of 0.0003 (Figure 3a–c). Moreover, after subtracting the optical density (OD) of water from the OD of the different sucrose solutions (Figure 3d–f), light intensity decreases and increases around 570 nm and 590 nm, respectively. Consequently, the OD change served as an output signal through subtraction of two wavelengths (OD_590_–OD_575_). The two-wavelength OD values correlated positively with the concentration of the sucrose solutions (R^2^ = 0.999).

### 3.3. Assessing Antibody Immobilization and Optimization of Testing Conditions

**Figure 4:**
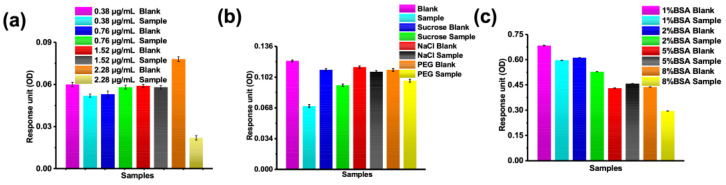
Optimization of antibody-labeling density and buffer composition. (**a**) Different concentrations of sulfamethazine antibody on the surface of gold nanoparticles. (**b**) Response unit measurements (optical density [OD]) for several reaction-promoting reagents in basic buffer. (**c**) Response unit measurements for different concentrations of bovine serum albumin in basic buffer.

Before conducting the competitive immunoassays for small-molecule detection using the MSPRAN biosensor, the AuNP antibody-labeling density and testing buffer were optimized. Different antibody labeling densities were evaluated for small-molecule detection, and a final concentration of 2.28 μg/mL sulfamethazine antibody labeled on the surface of AuNPs was found to clearly distinguish the blank and a test sample (Figure 4a). Thereafter, the basic buffer (20 mM tris, 20 mM EDTA, 0.5% TW20) was added to various reagents to improve the reaction response. However, the addition of basic buffer to all tested reagents did not show any improvement or interference (Figure 4b).

Next, different concentrations of BSA were incubated with basic buffer, and the addition of BSA appeared to improve the response unit significantly (approximately 6-fold) (Figure 4c). The reaction value with addition of 1% BSA was the highest and allowed for a clear distinction between the blank and the test sample. Therefore, an R1 buffer containing BSA (20 mM tris, 20 mM EDTA, 0.5% TW20, and 1%BSA) was selected for the competitive immunoassay.

### 3.4. Establishment of a Standard Curve for Small-Molecule Quantitative Analysis

**Figure 5:**
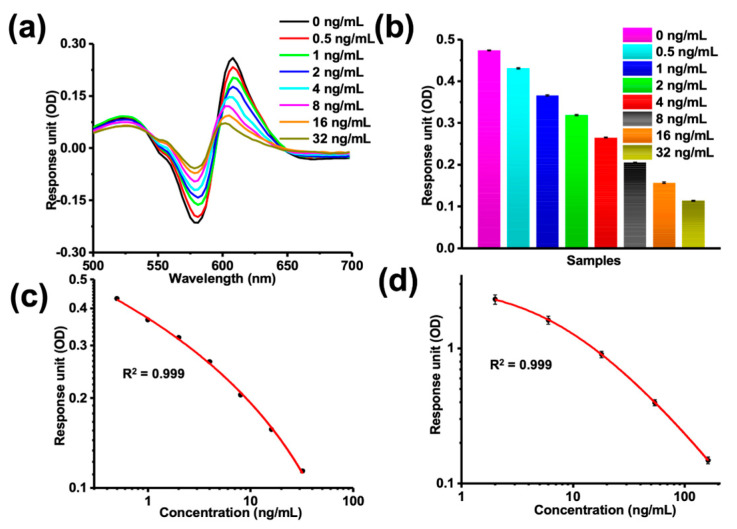
Sulfamethazine detection by a MetaSPR chip coupled with AuNPs (MSPRAN) biosensor and enzyme-linked immunosorbent assay (ELISA). (**a**) Response unit signals (optical density [OD]) measured for different concentrations of sulfamethazine (0–32 ng/mL). (**b**) Two-wavelength OD values (OD_608_–OD_581_) for different concentrations of sulfamethazine. (**c**) Standard curve of the two-wavelength OD values measured during sulfamethazine detection with MSPRAN (R^2^ = 0.999). (**d**) Standard curve for sulfamethazine detection using an ELISA kit (R^2^ = 0.999).

Sulfamethazine is a synthetic antibiotic with broad-spectrum antibacterial activity, good curative effect, and low cost and is widely used in the poultry industry, with residues in food posing health issues. Therefore, sulfamethazine was selected as a model drug for assessing the ability of the 96-well MSPRAN biosensor to detect small molecules. The MSPRAN biosensor can be detected by a general microplate reader or a WeSPR100 Multifunctional Molecular Analyzer (Shanghai, China), making it cost-effective and applicable to resource-poor areas and private laboratories. With an increase in sulfamethazine concentration, OD values increased at around 581 nm and decreased at around 608 nm wavelengths (Figure 5a). In addition, the two-wavelength OD values (OD_608_–OD_581_) decreased in a dose-dependent manner from 0 ng/mL to 32 ng/mL (Figure 5b). There was a good correlation between the concentration and these two-wavelength OD values according to a four-parameter logistic (4-PL) regression equation (R^2^ = 0.999; Figure 5c), and that equation can serve as a standard curve to calculate the limit of detection (LOD) value. The equation was as follows:

y=−0.36+1.711+x0.21^0.19

In addition, an ELISA kit was used to verify the MSPRAN biosensor by evaluating the correlation between a sulfamethazine concentration (0–162 ng/mL) and reaction signal (Figure 5d), which yielded R^2^ = 0.999, a result that was similar to the result obtained with the MSPRAN biosensor. These results showed that the MSPRAN biosensor could serve as a tool for the quantitative detection of sulfamethazine. Moreover, this result indicates that further studies are warranted to investigate the capabilities of this technique for real sample analysis.

### 3.5. MSPRAN Performance Evaluation for Sulfamethazine Detection

To verify the sensitivity of the MSPRAN biosensor, the LOD values were calculated from the standard curve according to methods described in a previous study [33]. The OD value of a blank sample minus three times the standard deviation was interpolated into the standard curve to obtain the corresponding concentration values, which served as the LOD value. The LOD for sulfamethazine detected by the MSPRAN biosensor was 73 pg/mL (Figure 5c). A comparison of different methods for sulfamethazine detection is shown in Table 1. LOD value of this study was lower than those of other detection methods, indicating that the MSPRAN biosensor was highly sensitive.

In addition, substrate selectivity was also assessed by testing different non-specific small molecules, such as chloramphenicol, enrofloxacin, ofloxacin, florfenicol, and fipronil, at a concentration of 50 ng/mL. None of the non-specific molecules tested exhibited a measurable reaction on the sulfamethazine detection platform (data not shown). In addition, repeatability was tested by performing measurements at different concentrations of sulfamethazine, and data were characterized as the coefficient of variation (CV) values. The CV values measured for various concentrations of sulfamethazine (2 ng/mL, 4 ng/mL, 16 ng/mL) were 7.07%, 5.63%, and 5.77%, respectively. Overall, these results showed that the 96-well MSPRAN biosensor displayed favorable performance during sulfamethazine detection.

### 3.6. Sulfamethazine Analysis in Whole Eggs

The practical application of the 96-well MSPRAN biosensor was evaluated using sulfamethazine-spiked whole eggs. Recovery rates of 87.29–119.30% were obtained for sulfamethazine detection using the MSPRAN biosensor, which were similar to the recovery rates obtained with ELISA (Table 2). The experimental steps of ELISA for sulfamethazine detection in whole eggs are shown in the Supplemental Materials. The consistency of recoveries between the 96-well MSPRAN biosensor and ELISA indicates that the biosensor could be used as a reliable alternative for small-molecule detection. In addition, the ELISA-based detection of sulfamethazine requires more than 1 h, while the detection with MSPRAN required only 10 min; thus, the MSRPAN approach facilitated fast measurements and high-throughput. Moreover, the operational steps of ELISA are complex, requiring five sample addition steps and multiple washing steps. However, our approach only required two sample addition steps and no washing steps, and can be performed following the manufacturer’s instructions, without specific training. Therefore, the developed MSPRAN biosensor was easy to operate. Taken together, the developed MSPRAN biosensor shows multiple advantages for small-molecule detection.

## 4. Conclusions

In this study, an MSPRAN biosensor for small-molecule detection was developed using a competitive immunoassay. The biosensor was designed to have low cost and portability, and demonstrated fast reads, high sensitivity, and high accuracy. The detection of sulfamethazine using the MSPRAN biosensor could be completed within only 10 min. Overall, our results demonstrated that the performance of the MSPRAN is comparable to that of the corresponding ELISA, a more widely used technique for small-molecule analysis. However, a limitation of the developed MSPRAN biosensor for sulfamethazine detection is that the antibody needs to be conjugated to the surface of AuNPs, which is a complicated and time-consuming operation. In the future, the sensitivity of the MetaSPR chip can be improved to detect small molecules directly by immobilizing antibodies on the chip surface. The novel MSPRAN biosensor for small-molecule detection developed here has not been reported previously and may pave the way for small-molecule detection based on a MetaSPR chip for use in food safety control and environmental monitoring. In addition, this approach holds potential for small/macro-molecule multiplexing analysis using a MetaSPR chip that is modified with multichannel plates with more than 96 wells. This could then be applied for large-scale food safety monitoring, environmental monitoring, and medical diagnoses.

## Figures and Tables

**Table 1:** Comparison of several detection methods for sulfamethazine.

Sample	Detection Method	Limit of Detection	Reference
Sulfamethazine	UPLC–MS/MS	0.14 μg/kg	[34]
	ELISA	0.19 ng/mL	[35]
	TLC	40 ng/g	[36]
	MSPRAN biosensor	73 pg/mL	This work

UPLC–MS/MS: Ultra-performance liquid chromatography–tandem mass spectrometry.

**Table 2:** Detection of sulfamethazine in whole eggs using the MSPRAN biosensor, in comparison with ELISA.

Concentration (ppb)	ELISA		MSPRAN	
	Average (ppb)	Recovery (%)	Average (ppb)	Recovery (%)
2	1.92 ± 0.03	96.20	1.75 ± 0.16	87.29
4	4.51 ± 0.07	112.60	3.92 ± 0.12	98.00
16	17.4 ± 006	109.30	19.1 ± 0.18	119.30

## Data Availability

The data that support the findings of this study are available in the Supplementary Materials of this article.

## Supplementary Materials

The following supporting information can be downloaded at: https://www.mdpi.com/article/10.3390/bios13070681/s1, Figure S1: Sulfamethazine detection of blank eggs using LC–MS; Figure S2: The standard curve of varies concentration of sulfamethazine (0–72 ppb) spiked in eggs according to response unit detected by the MetaSPR Biosensor; Figure S3: The standard curve of different concentration of sulfamethazine (0–162 ppb) spiked in eggs corresponding to reaction signal detected by an ELISA kit.
