# Supplementary material for: A Metasurface Plasmonic Analysis Platform Combined with Gold Nanoparticles for Ultrasensitive Quantitative Detection of Small Molecules
Source: Biosensors (Basel). 2023 Jun 27;13(7):681. doi: 10.3390/bios13070681 (PMC10377222; doi:10.3390/bios13070681)
Supplement: Supplementary file 1 [file biosensors-13-00681-s001.zip › biosensors-2328862-supplementary.pdf]

biosensors

MDPI

Supplementary Material

A Metasurface Plasmonic Analysis Platform Combined with Gold Nanoparticles for Ultrasensitive Quantitative Detection of Small Molecules

Taohong Zhou[1]; Weihao Ji[1]; Hongli Fan[2], Li Zhang[1,3,4]; Xugang Wan[1,3,4]; Zhiyong Fan[1,3,4]; Gang Logan Liu[2,*]; Qingzhi Peng[1,3,4,*]; Liping Huang [2,5,*]

1 Hubei Provincial Institute for Food Supervision and Test, Wuhan 430075, China

2 College of Life Science and Technology, Huazhong University of Science and Technology, Wuhan 430074, China

3 Key Laboratory of Detection Technology of Focus Chemical Hazards in Animal-derived Food for State Market Regulation, Wuhan 430075, China

4 Hubei Provincial Engineering and Technology Research Center for Food Quality and Safety Test, Wuhan 430075, China

5 Liangzhun (Wuhan) Life Technology Co. Ltd., 666 Gaoxin Avenue, Wuhan 430070, China

Sulfamethazine analysis in whole eggs using an ELISA kit

In order to evaluate the feasibility of using the metasurface plasmon resonance (MetaSPR) biosensor, the same real sample of whole eggs spiked with sulfamethazine that was analyzed with the MetaSPR biosensor was also studied using an ELISA kit. The process was performed according to the manufacturer’s instructions. A volume of 50 μL each of standard product and sample was added to the corresponding microwells. Then, 50 μL of sulfamethazine antibody was added to the microwell, followed by gentle shaking. After the plate was covered with film to shield it from light, the reaction was carried out at 37 °C for 30 min. Then, after peeling off the cover film, the liquid in the well was discarded, after which 250 μL washing liquid was added to the microwell. After letting this stand for 20 s, the liquid in the well was removed and discarded. This process was repeated three times. Labeled sulfamethazine enzyme (100 μL) was added to each well, and the reaction was allowed to proceed in a dark environment, shaking, at 37°C for 30 min. Then, the plate was washed three times, as previously described. Substrate solution A was added to each well (50 μL), followed by the same amount of substrate solution B. The plate was again covered with film and shaken at 37°C for 15 min. Then, 50 μL of termination solution was added to each well, and the absorbance value was immediately measured at 450 nm using a microplate reader.

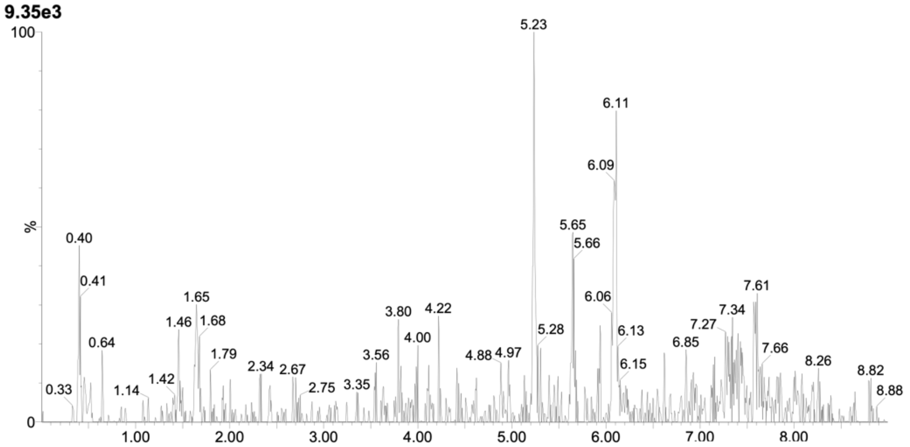

Figure S1. Sulfamethazine detection of blank eggs using LC–MS.

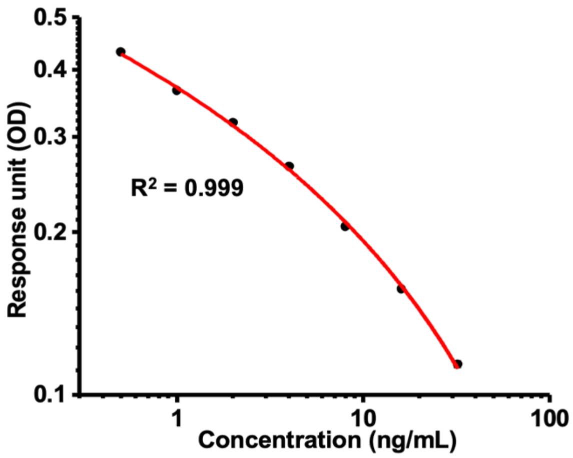

Figure S2. The standard curve of varies concentration of sulfamethazine (0-72 ppb) spiked in eggs according to response unit detected by the MetaSPR Biosensor.

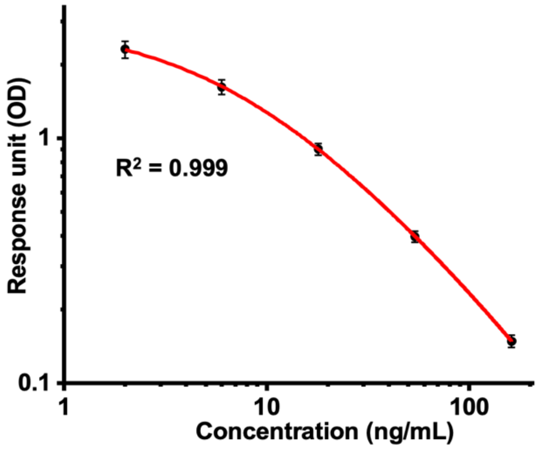

Figure S3. The standard curve of different concentration of sulfamethazine (0-162 ppb) spiked in eggs corresponding to reaction signal detected by an ELISA kit.
